# Nicotinamide Supplementation Improves Oocyte Quality and Offspring Development by Modulating Mitochondrial Function in an Aged *Caenorhabditis elegans* Model

**DOI:** 10.3390/antiox10040519

**Published:** 2021-03-26

**Authors:** Hyemin Min, Mijin Lee, Kyoung Sang Cho, Hyunjung Jade Lim, Yhong-Hee Shim

**Affiliations:** 1 Department of Bioscience and Biotechnology, Konkuk University, Seoul 05029, Korea; mintmin@konkuk.ac.kr (H.M.); miranda12@konkuk.ac.kr (M.L.); 2 Department of Biological Sciences, Konkuk University, Seoul 05029, Korea; kscho@konkuk.ac.kr; 3 Department of Veterinary Medicine, Konkuk University, Seoul 05029, Korea; hlim@konkuk.ac.kr

**Keywords:** nicotinamide, antioxidant, reproductive aging, oocyte quality, mitochondrial function, reactive oxygen species (ROS), *C. elegans*

## Abstract

Aging is associated with a decline in the quality of biological functions. Among the aging processes, reproductive aging is a critical process because of its intergenerational effects. However, the mechanisms underlying reproductive aging remain largely unknown. Female reproductive aging is the primary reason for limited fertility in mammals. Therefore, we attempted to investigate a modulator that can control female reproductive aging using a *Caenorhabditis elegans* model. In the present study, we examined the role of nicotinamide (NAM) in oocyte quality and offspring development. The levels of reactive oxygen species (ROS) and oxidative stress responses in aged oocytes, embryonic lethality, and developmental growth of the offspring were examined with maternal NAM supplementation. Supplementation with NAM improved oocyte quality, decreased embryonic lethality, and promoted germ cell apoptosis. Furthermore, NAM supplementation in aged mothers reduced ROS accumulation and improved mitochondrial function in oocytes. Consequently, the developmental growth and motility of offspring were improved. These findings suggest that NAM supplementation improves the health of the offspring produced by aged mothers through improved mitochondrial function. Taken together, our results imply that NAM supplementation in the aged mother improves oocyte quality and protects offspring by modulating mitochondrial function.

## 1. Introduction

Female reproductive capacity rapidly declines after the mid-30s, which is accompanied by an increased risk of infertility, miscarriage, embryonic lethality, and birth defects in humans [1,2]. Reduction in female reproductive capacity with aging is mainly due to the deterioration of oocyte quality [3,4]. As the age of pregnancy in modern society increases, understanding the basis of female reproductive aging has become an important issue for fertility. In particular, observations of changes in aged oocytes in animal model organisms have emerged [5,6].

*Caenorhabditis elegans* has been developed as an excellent animal model for studying reproductive capacity with age [7,8,9]. Previous studies have demonstrated that a genetically regulated process of reproductive aging is limited by oocyte quality. In the germline of *C. elegans* hermaphrodites, germ cell development can be observed from germ cell proliferation, spermatogenesis, oogenesis, and germ cell death [10]. Additionally, *C. elegans* oocytes can be identified with age in a stage-specific manner; therefore, aged oocytes can be efficiently observed at certain time points. Both mammalian and *C. elegans* oocytes are arrested at prophase I during oogenesis, which are then released by hormonal control. The released oocytes subsequently undergo maturation [11,12]. The process of oocyte maturation is highly conserved from worms to humans in which oocyte quality is the major factor for reproductive capacity. Therefore, it is worthwhile to investigate female reproductive aging and the possible modulators during this process using aged oocytes in a *C. elegans* model.

Nicotinamide adenine dinucleotide (NAD^+^) is an important metabolite of vitamin B_3_ that plays an essential role in numerous biological processes [13]. Recent studies have demonstrated that NAD^+^ is a key redox cofactor that regulates diverse physiological processes, such as DNA repair, autophagy, stress response, genomic stability, and cell survival [14,15,16,17,18]. The level of NAD^+^ decreases with age in many tissues, which is associated with a variety of aging-associated diseases [19,20,21]. Although substantial progress has been made in the beneficial effects of the NAD+ precursor, nicotinamide mononucleotide (NMN) on aging, the effect of NMN on female reproductive aging has not been fully studied. In mice, ovulation occurs every 3–4 days and the length of gestation is about 20 days. Reproductive aging is thought to begin around the age of 8 months [22]. Oocyte quality declines around this time, with a heightened rate of chromosomal abnormalities and other cellular changes [5]. The role of nicotinamide adenine dinucleotide (NAD+) in oocyte aging has recently been suggested in the mouse model. This study showed that NAD+ levels decrease in oocytes from aged mice and that its repletion rescues certain aspects of reproductive capacity [5,6]. NAD+ precursor supplementation has beneficial effects on reproductive aging, which improves the age-dependent decline in oocyte quality, fertility, and embryo development in aged mice [5,6]. Although the effect of nicotinamide (NAM) on *Drosophila*, which is also a well-established animal model, reproductive aging is not well known, the nutritional supplementation of NAM decreased reactive oxygen species (ROS) levels and increased lifespan in aged flies [23] and prevented oxidative mitochondrial dysfunction [24]. Considering the important contribution of ROS on *Drosophila* reproductive aging, NAM can be expected to have a beneficial effect on reproductive aging in *Drosophila* as well.

In this study, we investigated the effects of NAM supplementation on the reproductive capacity of aged *C. elegans* oocytes and subsequent offspring development. Here, we report that age-related mitochondrial dysfunction in aged oocytes was improved by NAM supplementation. Furthermore, the mitochondrial function and oxidative stress resistance of offspring produced by NAM-treated aged mothers were also improved. Taken together, maternal NAM supplementation increased mitochondrial function and stress resistance in the offspring intergenerationally by promoting oocyte quality in an aged mother.

## 2. Materials and Methods

### 2.1. Caenorhabditis elegans Strains and Nicotinamide (NAM) Supplementation

*C. elegans* strains were maintained at either 15 or 20 °C on nematode growth medium (NGM) agar plates seeded with *Escherichia coli* strain OP50, as previously described [25]. The following strains were used in this study: N2 (*C. elegans* wild isolate, Bristol variety), YHS54: *opIs219 [ced-4p::ced-4::GFP]?*, MT1743: *ced-3(n718) IV*, TJ375: *gpIs1 [hsp-16.2p::GFP]*, CF1553: *muIs84 [(pAD76) sod-3p::GFP + rol-6(su1006)]*, SJ4005: *zcIs4 [hsp-4::GFP] V*, SJ4100: *zcIs13[hsp-6::GFP]*, SJ4103: *zcIs14 [myo-3::GFP(mit)]*, RW1596: *myo-3(st386) V; stEx30 [myo-3p::GFP::myo-3 + rol-6(su1006)]*. To examine the effect of NAM supplementation, 1 mM NAM (Sigma-Aldrich, St. Louis, MO, USA) was added to NGM before autoclaving. Young (synchronized 48 h post L4-stage animals) and aged (synchronized 96 h post L4-stage animals) mothers and their progenies were examined. For the NAM+aged group, the synchronized 72 h post L4-staged mothers were exposed to NAM and examined after 24 h incubation at 20 °C. For the experimental design, see Supplementary Figure S1.

### 2.2. Analysis of Embryonic Lethality, Unfertilized Oocytes, Small Embryos, and Percent Larval Development

The 72 h post-L4-stage wild-type N2 hermaphrodites were individually cloned onto either NAM-containing or NAM-free NGM agar plates and grown at 20 °C. They were transferred to new plates for 24 h to allow for embryo production. Laid embryos were considered dead if they did not hatch after 24 h at 20 °C. Embryonic lethality was calculated as the percentage of non-hatched embryos of the total number of embryos produced for 24 h at 20 °C. Unfertilized oocytes and small eggs were calculated as the number of either unfertilized oocytes or small eggs per mother. The percent larval development was calculated as the percentage of larvae of the total number of hatched embryos that reached each developmental stage, as previously described [26]. The developmental larval stages were distinguished as follows: L1, the smallest larvae (<0.3 mm); L2, larvae larger than L1 (body length, 0.3–0.4 mm) but with no characteristics of L3; L3, larvae with a white spot in the vulva region (body length, 0.4–0.6 mm); L4, larvae with a characteristic half-moon-like shape in the vulva region (body length, 0.6–0.8 mm); adults, animals with an opened vulva with eggs in the uterus.

### 2.3. DNA Staining in Oocytes

To observe whether an aged oocyte has six pairs of aligned condensed chromosomes (bivalents), DNA staining was performed as previously described [27]. Animals were dissected to extrude gonads in 10 µL of M9 buffer containing 100 µg/mL tetramisole on a poly L-lysine-coated slide, covered with a coverslip, freeze-cracked with liquid nitrogen, and fixed with methanol and acetone. The specimens were then stained with 1 µM TO-PRO-3 (Molecular Probes, Eugene, OR, USA) for 1 h at 20 °C and observed under a fluorescence microscope (Zeiss Axioscope, Oberkochen, Germany).

### 2.4. Immunofluorescence Analysis

Immunofluorescence analysis was performed as previously described [27]. Briefly, the animals were dissected to extrude gonads in 10 µL of M9 buffer containing 100 µg/mL tetramisole on a poly L-lysine-coated slide, covered with a coverslip, freeze-cracked with liquid nitrogen, and fixed with methanol and acetone. The gonads were then immunostained with primary and secondary antibodies. The specimens were counterstained with 0.5 mg/mL Hoechst 33342 dye to detect DNA and were observed under a fluorescence microscope (Zeiss Axioscope, Oberkochen, Germany). The primary antibodies were rabbit anti-GFP used for the detection of CED-4::GFP, the Apaf-1–like cell-death activator (1:400; Novus, Novus, Centennial, CO, USA) and mouse anti-RAD-51, an indicator of double-strand breaks (DSBs) (1:500; Novus, Centennial, CO, USA). Secondary antibodies were anti-rabbit IgG (Alexa Fluor 488 conjugated; 1:500; Invitrogen, Carlsbad, CA, USA) and anti-mouse IgG (Alexa Fluor 488 conjugated; 1:500; Invitrogen, Carlsbad, CA, USA).

### 2.5. Western Blot Analysis

Western blot analysis was performed as described previously [28]. Whole animal protein extracts were obtained from 300 gravid adult hermaphrodites of each condition per gel well. Antibodies bound to a nitrocellulose membrane (PROTRAN BA83, Whatman, Sigma-Aldrich, St. Louis, MO, USA) were visualized with an ECL Western blot detection kit (Amersham, GE Healthcare Life Sciences, Pittsburgh, PA, USA), and the band intensities were measured with the LAS-3000 image analyzer using Multi Gauge software (v.3.0, Fuji Film, Tokyo, Japan). The following primary antibodies were used: rabbit anti-CED-9, a member of the anti-apoptotic Bcl-2 gene family in *C. elegans* (1:1000; Santa Cruz Biotechnology, Dallas, TX, USA) and goat anti-CED-4, the pro-apoptotic Apaf-1–like cell-death activator (1:1000; Santa Cruz Biotechnology, Dallas, TX, USA). The mouse anti-α-tubulin was used as the loading control (1:1000; Sigma-Aldrich, St. Louis, MO, USA). The following secondary antibodies were used: horseradish peroxidase (HRP)-conjugated goat anti-rabbit IgG (1:1000; Santa Cruz Biotechnology, Dallas, TX, USA), HRP-conjugated donkey anti-goat IgG (1:1000; Santa Cruz Biotechnology, Dallas, TX, USA), and HRP-conjugated donkey anti-mouse IgG (1:1000; Jackson ImmunoResearch, West Grove, PA, USA).

### 2.6. Germ Cell Apoptosis Assay

Apoptotic germ cells were visualized by acridine orange (AO) vital staining, as previously described [29]. Briefly, the animals were stained with 25 µg/mL AO in M9 buffer for 1 h in the dark and then allowed to recover on new NGM plates seeded with OP50 for 20 min in the dark. The number of AO-positive germ cells per gonad arm was counted under a fluorescence microscope (Zeiss Axioscope, Oberkochen, Germany).

### 2.7. Analysis of Mitochondrial Activity and Mitochondrial Membrane Potential (MMP)

The animals were incubated for 4 h at 20 °C with 10 μM MitoTracker Red (Invitrogen, Carlsbad, CA, USA), which is a fluorescent probe that accumulates in active mitochondria. After MitoTracker staining, the animals were immobilized in 10 µL of M9 buffer containing 100 µg/mL tetramisole on a poly L-lysine-coated slide, and the animals were dissected for oocyte isolation. Live images of stained animals were observed under a fluorescence microscope (Zeiss Axioscope, Oberkochen, Germany), and the average pixel intensity of MitoTracker Red fluorescence was measured using ImageJ software. To measure MMP, tetramethylrhodamine methyl ester (TMRM; Thermo Fisher Scientific, Waltham, MA, USA) staining was performed, as previously described [30]. Briefly, TMRM was added to NGM agar at a final concentration of 30 μM, and the plates were dried overnight and seeded with *E. coli* OP50 for 24 h in the dark. Animals from each group (young, aged, and NAM+aged) were transferred to TMRM plates, incubated at 20 °C for 15 h, and dissected for oocyte isolation. For whole-body animal staining, each group of mothers was incubated in TMRM plates for 15 h. Images were obtained under the same exposure, and the fluorescence intensity was measured using ImageJ software by selecting oocytes and whole animals.

### 2.8. Analysis of Mitochondrial Reactive Oxygen Species (ROS)

To examine the effect of maternal NAM supplementation on mitochondrial ROS, CellROX^®^ Green (Invitrogen, Carlsbad, CA, USA) staining was performed as previously described [28]. CellROX^®^ Green is a fluorogenic probe for measuring oxidative stress in live cells. This cell-permeant dye exhibits bright green fluorescence upon oxidation by ROS and subsequent binding to mitochondrial DNA. Briefly, CellROX^®^ Green was freshly prepared in a 5 mM stock solution and diluted in M9 buffer at a 1:1000 dilution before treatment. The animals were transferred into a staining solution and stained for 20 min at 20 °C. The animals were mounted on a poly L-lysine-coated slide and observed under a fluorescence microscope (Zeiss Axioscope, Oberkochen, Germany). The relative quantitation of mitochondrial ROS was performed using ImageJ software.

### 2.9. Live Image Observation of Fluorescence-Tagged Transgenic Animals

To observe the expression of HSP-6 and SOD-3 in the F1 generation by maternal NAM supplementation, transgenic strains SJ4100 and CF1553 were observed. SJ4100, *zcIs13 [hsp-6::GFP]*, contains a *hsp-6::GFP* transgene as a marker of mitochondrial unfolded protein response in which the *GFP* gene is driven by the *hsp-6* promoter. CF1553, *muIs84 [sod-3::GFP]*, contains a *sod-3::GFP* transgene as a marker of oxidative stress response in which *GFP* gene is driven by the *sod-3* promoter. To investigate the mitochondrial morphology and integrity of muscle cells in the F1 generation by maternal NAM supplementation, transgenic strains SJ4103 and RW1596 were observed, respectively. SJ4103 contains a transgene *myo-3::GFP(mit)*, which restricts the expression of GFP to the mitochondria of body muscle tissue and RW1596 contains a transgene *[myo-3p::GFP::myo-3]*, expressing a GFP-tagged muscle myosin. The F1 generation animals produced by each group expressing GFP were immobilized in 10 µL of M9 buffer containing 100 µg/mL tetramisole on a poly L-lysine-coated slide. Live images of the animals were observed under a fluorescence microscope (Zeiss Axioscope, Oberkochen, Germany).

### 2.10. Locomotion Behavior Assay

Locomotion behavior was analyzed by monitoring head thrashing and body bending, as previously described [31]. Briefly, to assay head thrashing, the F1 generation animals that were produced by each group of mothers were washed with M9 buffer and transferred to a plate containing 100 µL of M9 buffer on non-food NGM agar. The head thrashes were counted for 1 min. To assay body bending, the animals were transferred to a new plate and scored for the number of body bends at 20 s intervals. One body bend was defined as a complete cycle of terminal bulb motion, starting from the top position of the sinusoidal wave track through to the bottom and back to the top.

### 2.11. Survival Assay under Paraquat-Induced Oxidative Stress

Paraquat survival assay was performed as previously described with minor modifications [32]. In brief, to analyze the survival rate under oxidative stress conditions in each group of the F1 generation, the synchronized L1 larval stage animals were exposed to 100 mM paraquat solution for 6 h at 20 °C, and then dead and live animals were counted. The animals were considered dead when they failed to respond to a gentle touch with a platinum wire on their bodies.

### 2.12. Statistical Analysis

All experiments were repeated more than three times for the statistical evaluation of the data. The *p* values were calculated using either a two-tailed Student’s *t*-test or ANOVA with Tukey’s post hoc test. Values with *p* < 0.05 were considered statistically significant. Data are expressed as the mean ± standard deviation (SD). Statistical analyses were performed using jamovi software.

## 3. Results

### 3.1. Nicotinamide (NAM) Supplementation Improves Fertility of Aged Oocytes

**Figure 1:**
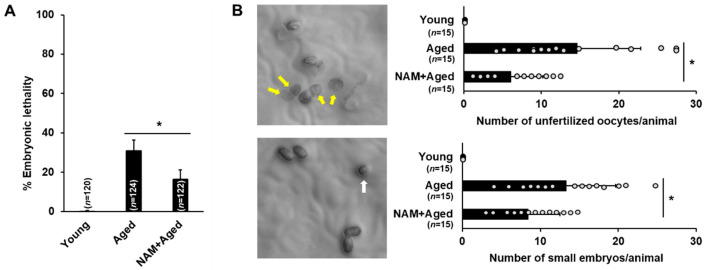
The fertility of aged oocytes is improved by nicotinamide (NAM) supplementation in *Caenorhabditis elegans*: (**A**) The percentage of embryonic lethality among the total number of progenies produced by young, aged, and NAM-treated aged (NAM+aged) N2 hermaphrodites. Error bars represent standard deviation (SD). *, *p* < 0.05 (one-way ANOVA with Tukey’s post hoc test); (**B**) The number of unfertilized oocytes (upper graph) and small embryos (bottom graph) among the total number of progenies of young, aged, and NAM+aged N2 hermaphrodites. Representative images of unfertilized oocytes (indicated by yellow arrows) and a small embryo (indicated by a white arrow). Error bars represent SD. *, *p* < 0.05 (one-way ANOVA with Tukey’s post hoc test).

The decline in oocyte quality is associated with age-related infertility. Therefore, issues for the maintenance of oocyte quality in an aged mother are emerging, hoping to find a way to delay reproductive aging. Nicotinamide adenine dinucleotide (NAD^+^) improves oocyte quality during reproductive aging in a mouse model [5,6]. Due to the advantages of the *C. elegans* model for studying oocyte quality and the subsequent offspring development, we investigated the effects of maternal NAM supplementation in the present study. We attempted to measure embryonic lethality (EL) as an indicator of oocyte quality after supplementation with NAM in an aged mother. First, EL was measured after maternal NAM supplementation in *C. elegans* at concentrations of 0.1, 0.5, 1, 5, 10, and 50 mM (Supplementary Figure S2) to determine the optimal conditions for the study. We found that NAM supplementation significantly decreased EL at 1 mM NAM (Supplementary Figure S2 and Figure 1A) and that concentrations higher than 1 mM resulted in deteriorating effects on worms. Therefore, the test animals were treated with 1 mM NAM in this study.

To examine the effect of NAM supplementation on the infertility of aged animals, the number of unfertilized oocytes and small embryos was counted (Figure 1B). Compared to non-treated mothers, NAM-treated mothers produced significantly fewer unfertilized oocytes and defective embryos (Figure 1B). These results suggest that NAM supplementation decreases embryonic lethality through improved fertilization and embryo morphology.

### 3.2. NAM Supplementation Improves Chromosomal Abnormalities in Aged Oocytes and Decreased Level of Germ Cell Apoptosis in Aged Animals

**Figure 2:**
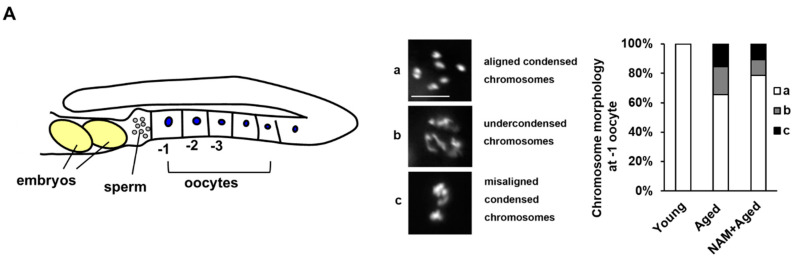

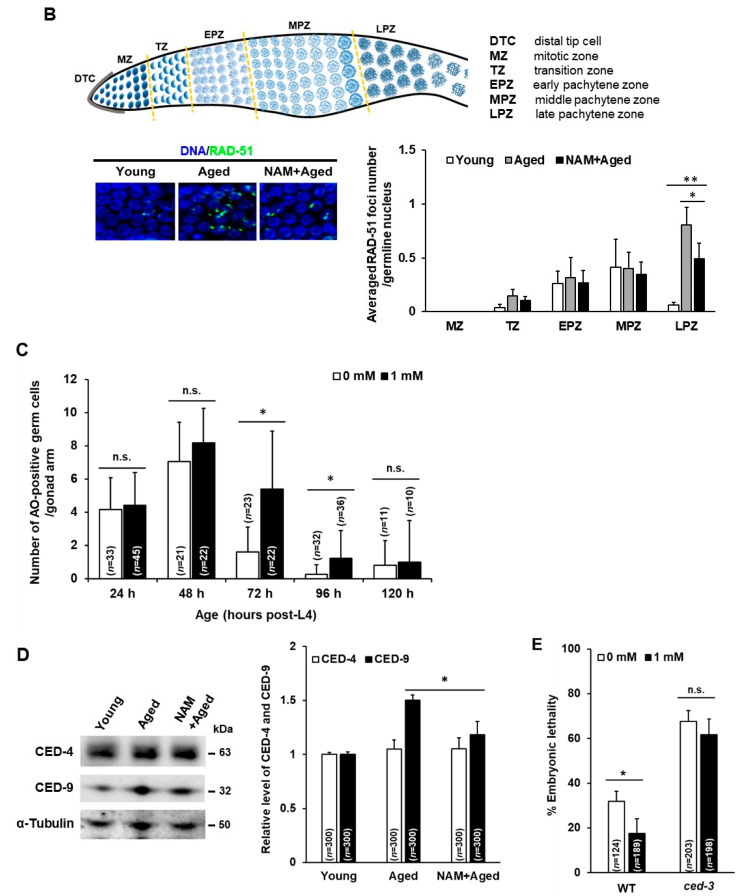
Chromosomal abnormalities in aged oocytes and the decreased germ cell apoptosis level in an aged mother are improved by nicotinamide (NAM) supplementation in *Caenorhabditis elegans*: (**A**) Schematic illustration of oocytes in the *C. elegans* hermaphrodite germline. Chromosomal aberrations were observed by DNA staining in young, aged, and NAM+aged at -1 oocytes. The type of aberration was classified into three categories: (1) aligned and condensed chromosomes, (2) undercondensed chromosomes, and 3) misaligned and condensed chromosomes. The percent distributions of the respective categories in young, aged, and NAM+aged oocytes are presented; (**B**) Schematic illustration of each zone in *C. elegans* germline. Representative images of the RAD-51 foci (green) in the late pachytene region are shown. The numbers of RAD-51 foci per germ nucleus in each group are shown (each condition, *n* = 30). Error bars represent standard deviation (SD). *, *p* < 0.05, **, *p* < 0.01 (two-way ANOVA with Tukey’s post hoc test); (**C**) Average numbers of acridine orange (AO)-positive germ cells per gonad arm in non-treated (0 mM) or NAM-treated (1 mM) hermaphrodites with age. Error bars represent SD. n.s., not significant, *, *p* < 0.05 (two-way ANOVA with Tukey’s post hoc test); (**D**) Western blot analysis of CED-4 and CED-9 protein levels using anti-CED-4 and anti-CED-9 antibodies in each condition. α-tubulin was used as a loading control. Relative expression levels of CED-4 and CED-9 in each condition are shown. CED-4 or CED-9 band intensity was normalized to that of α-tubulin on the same lane, and the relative levels of CED-4 and CED-9 were converted to a relative value against that of the young mothers as 1. Error bars represent SD. n.s., not significant, *, *p* < 0.05 (two-way ANOVA with Tukey’s post hoc test); (E) The percentage of embryonic lethality among the total number of progenies produced by either aged wild-type (WT) N2 or aged *ced-3* mutants supplemented with or without 1 mM NAM. Error bars represent SD. *, *p* < 0.05, n.s., not significant (two-way ANOVA with Tukey’s post hoc test).

Increased errors during chromosome segregation are associated with maternal age-related miscarriages [33]. Chromosomal abnormalities are a major cause of mammalian embryonic developmental defects [34,35,36]. To determine whether NAM supplementation affects chromosomal integrity in aged oocytes, we observed DAPI (4′,6-diamidino-2-phenylindole)-stained chromosomes in the oocytes of both non-treated and NAM-treated animals. The number of oocytes with a normal number of stained chromosomes (six bivalents, aligned condensed chromosomes) decreased significantly with age in the wild type, which was improved by NAM supplementation (Figure 2A and Table 1). These results suggest that the chromosomal integrity in aged oocytes is better maintained with NAM supplementation.

Double-strand breaks (DSBs), which occur during germ cell differentiation to facilitate chromosomal crossing over, are repaired by homologous recombination (HR) [37,38,39]. However, this process becomes defective with aging, and DSBs remain not fully repaired [37]. The remaining DSBs can be detected by immunostaining of RAD51, which binds at the site of DSBs [40]. To determine whether the number of DSBs was increased in aged oocytes, we performed immunostaining against RAD-51 foci with or without NAM supplementation (Figure 2B). We found a significantly increased number of RAD-51 foci in the late pachytene zone with age. Strikingly, the increased number of RAD-51 foci was decreased by NAM supplementation (Figure 2B). These results suggest that NAM supplementation contributes to maintaining chromosomal integrity in aged oocytes by decreasing the level of RAD-51 foci.

Germ cell apoptosis in *C. elegans* maintains oocyte quality in adult stage-animals by reallocating resources for proper reproduction [7,10]. However, the level of germ cell apoptosis decreases with age when fertility is reduced [41]. To determine whether NAM supplementation controls germ cell apoptosis in aged animals to sustain the reproductive period, we measured the germ cell apoptosis level after NAM supplementation in aged animals (Figure 2C). As expected, the level of germ cell apoptosis was highest at 48 h after which it decreased dramatically. However, the germ cell apoptosis level was significantly increased after 72 and 96 h of NAM supplementation. These findings suggest that increased germ cell apoptosis by NAM supplementation improves oocyte quality in aged animals. Furthermore, the increased level of germ cell apoptosis by NAM supplementation was dependent on the decrease in the level of CED-9, a homolog of anti-apoptotic Bcl-2 [42] but not on that of CED-4, a homolog of pro-apoptotic Apaf-1 [42] (Figure 2D and Supplementary Figure S3), suggesting that the anti-apoptotic CED-9 is the primary target controlling germ cell apoptosis in aged animals. Additionally, the CED-9 level was decreased by NAM supplementation, suggesting that the increased germ cell apoptosis level in aged animals with NAM supplementation was due to the decreased CED-9 level. Finally, as NAM increased the germ cell apoptosis level and oocyte quality in aged animals, we examined the effect of NAM supplementation on embryonic lethality in both wild-type and apoptosis-defective *ced-3* mutants (Figure 2E). Embryonic lethality was significantly decreased in wild-type mothers by NAM supplementation but not in *ced-3* mutants (Figure 2E). Together, our results suggest that NAM improves oocyte quality by maintaining chromosomal integrity in aged oocytes and germ cell apoptosis in aged animals, which consequently reduces embryonic lethality.

### 3.3. NAM Supplementation Improves Mitochondrial Dysfunction in Aged Oocytes

**Figure 3:**
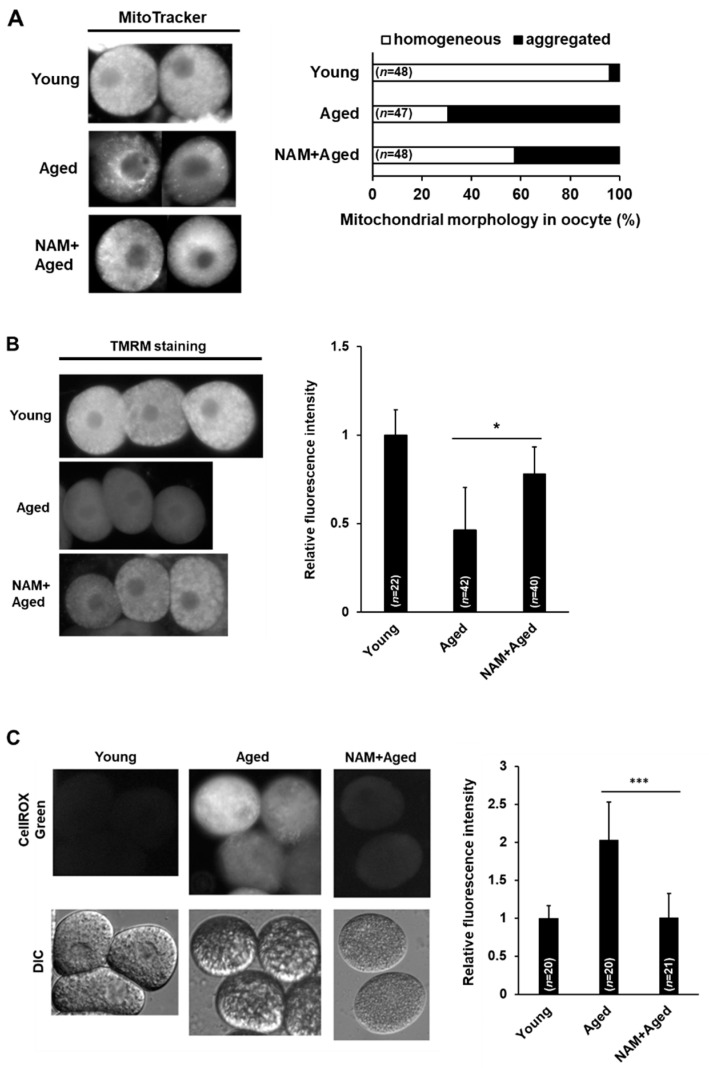
Mitochondrial dysfunction in aged oocytes is improved by nicotinamide (NAM) supplementation in *Caenorhabditis elegans*: (**A**) Comparison of mitochondrial activity and morphology in young, aged, and NAM+aged oocytes using MitoTracker staining. The graph indicates the percentage of oocytes with mitochondria classified as homogeneous or aggregated; (**B**) Comparison of mitochondrial membrane potential (MMP) in young, aged, and NAM+aged oocytes using tetramethylrhodamine methyl ester (TMRM) staining. The fluorescence of TMRM was quantified for each condition using ImageJ. The graph shows the relative levels of MMP. Error bars represent standard deviation (SD). *, *p* < 0.05 (one-way ANOVA with Tukey’s post hoc test); (**C**) Comparison of mitochondrial reactive oxygen species (ROS) levels in young, aged, and NAM+aged oocytes using CellROX Green staining. The graph shows the relative levels of mitochondrial ROS by ImageJ analysis. Error bars represent SD. ***, *p* < 0.001 (one-way ANOVA with Tukey’s post hoc test).

Abnormal mitochondrial membrane potential (MMP) affects germ cell apoptosis in *C. elegans* [30]. Furthermore, mitochondrial dysfunction is one of the prominent phenotypes observed in aged oocytes as previously reported in a mouse model [5]. Therefore, we examined mitochondrial function in aged oocytes after NAM supplementation by observing the distribution of mitochondria using MitoTracker staining and MMP using TMRM staining. Aggregated mitochondrial distribution in the cytoplasm with reduced intensity was observed in aged oocytes, while a homogeneous distribution of mitochondria in the cytoplasm was observed in the majority of young oocytes (Figure 3A). The aggregated mitochondrial distribution in aged oocytes was significantly reduced by NAM supplementation (Figure 3A).

MMP is essential for mitochondrial functions, such as ATP production and mitochondrial dynamics, including fusion and fission events for survival [43,44]. Therefore, we further examined the MMP by TMRM staining in aged oocytes with or without NAM supplementation. We measured the intensity of TMRM fluorescence in oocytes and the body with or without NAM supplementation in aged animals (Figure 3B). The MMP was significantly reduced in aged oocytes compared to that in young oocytes (Figure 3B). In contrast, no significant change was observed in the MMP in the body at this time, suggesting that somatic aging has not yet progressed (Supplementary Figure S4). Furthermore, the decreased TMRM fluorescence level was improved by NAM supplementation in aged oocytes (Figure 3B). These results suggest that NAM improves the MMP in aged oocytes.

It is well known that mitochondrial dysfunction is a major cause of reactive oxygen species (ROS) generation; therefore, we measured ROS levels and examined the effect of NAM supplementation on ROS generation in aged oocytes using CellROX Green, a fluorogenic probe for measuring mitochondrial ROS in live cells. The fluorescence intensity was significantly stronger in aged oocytes than in young oocytes (Figure 3C). Additionally, aged oocytes supplemented with NAM showed significantly reduced ROS levels (Figure 3C). This result supports the previous finding that the reduced MMP observed in aged oocytes was restored by NAM supplementation.

### 3.4. Maternal NAM Supplementation Improves Mitochondrial Activity and Oxidative Stress Responses in Offspring Produced by Aged Oocytes

**Figure 4:**
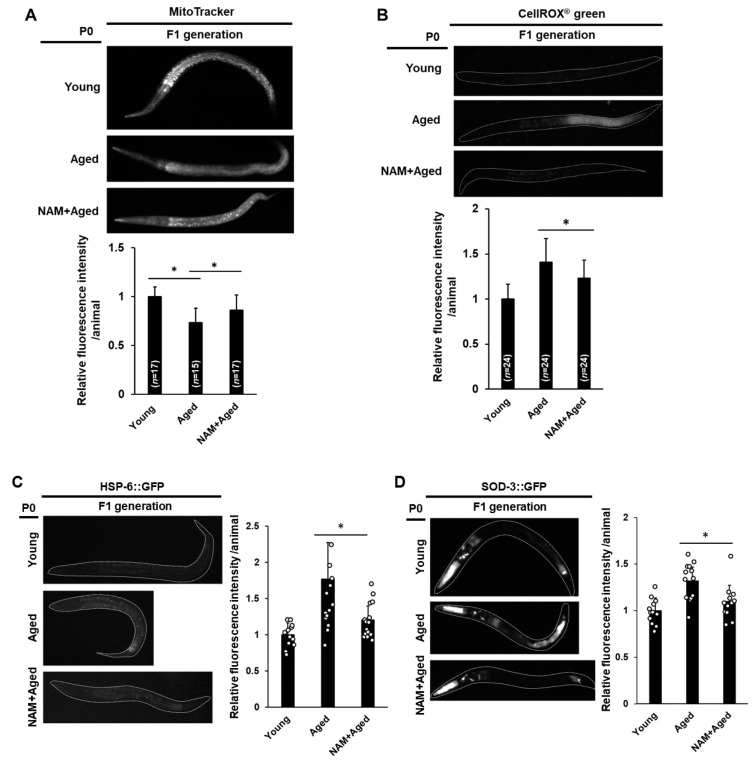
Mitochondrial activity and oxidative stress responses in offspring produced by aged oocytes are improved by maternal nicotinamide (NAM) supplementation in *Caenorhabditis elegans*: (**A**) Comparison of mitochondrial activity in the F1 generation produced by young, aged, and NAM+aged mothers using MitoTracker staining. The graph shows the relative levels of mitochondrial activity by ImageJ analysis. Error bars represent standard deviation (SD). *, *p* < 0.05 (one-way ANOVA with Tukey’s post hoc test); (**B**) Comparison of mitochondrial reactive oxygen species (ROS) levels in the F1 generation produced by young, aged, and NAM+aged mothers using CellROX Green staining. The graph shows the relative levels of mitochondrial ROS by imageJ analysis. Error bars represent SD. *, *p* < 0.05 (one-way ANOVA with Tukey’s post hoc test); (**C**,**D**) The F1 generation of HSP-6::GFP and SOD-3::GFP transgenic animals produced by young, aged, and NAM+aged mothers, and respective fusion proteins were observed under fluorescence microscopy and quantified using ImageJ (each condition, *n* = 20). Error bars represent SD. *, *p* < 0.05 (one-way ANOVA with Tukey’s post hoc test).

Optimal mitochondrial function is important for oocyte and embryo development [45]. Considering that maternal mitochondria are exclusively transmitted to offspring, dysfunctional mitochondria from aged oocytes may lead to developmental abnormalities in the offspring. Therefore, we examined the possible beneficial effects of maternal NAM supplementation on offspring through improved mitochondrial function in aged oocytes. We first observed the mitochondrial activity of the offspring produced from aged mothers by measuring MitoTracker fluorescence. The mitochondrial activity in the F1 generation from aged mothers was significantly reduced compared to that in the F1 generation from young mothers (Figure 4A). However, the F1 generation from NAM-treated aged mothers showed increases in mitochondrial activity compared to the F1 generation from aged non-treated mothers (Figure 4A), suggesting that dysfunctional mitochondria transmitted from aged animals to the next generation were improved by maternal NAM supplementation. We further investigated whether there was an alteration in ROS levels in the F1 generation produced by aged mothers with or without maternal NAM supplementation. Therefore, we measured the level of ROS in the F1 generation using CellROX Green. While the intensity was significantly stronger in the F1 generation from aged mothers than that from young mothers, the F1 generation from NAM-treated aged mothers showed a significantly lower level of ROS compared to the F1 generation from non-treated aged mothers (Figure 4B). Furthermore, we confirmed the significant induction of the mitochondrial stress response reporter, HSP-6, and the ROS detoxification enzyme, SOD-3, in the F1 generation from aged mothers through the observation of transgenic animals (Figure 4C,D). We found that maternal NAM supplementation suppressed the elevated levels of HSP-6 and SOD-3 in the F1 generation compared to that in the F1 generation from non-treated aged mothers (Figure 4C,D). We also found no alterations in the expression of an endoplasmic reticulum chaperone (HSP-4) and a cytosolic chaperone (HSP-16.2) when observed in each transgenic animal (Supplementary Figure S5). These findings indicate that maternal dysfunction in the mitochondria of aged animals is at least partially responsible for regulating the expression of the mitochondrial stress response and ROS detoxification in the offspring.

### 3.5. Maternal NAM Supplementation Improves Motility, Oxidative Stress Resistance, and Developmental Growth in Offspring Produced by Aged Oocytes

**Figure 5:**
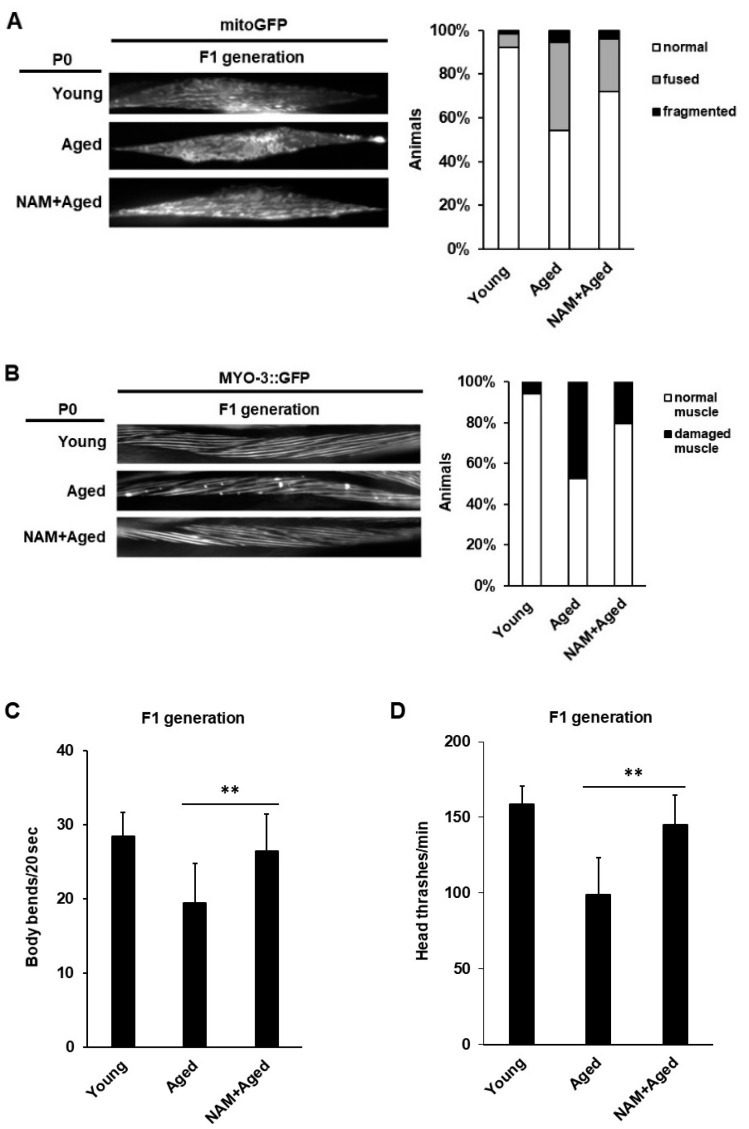

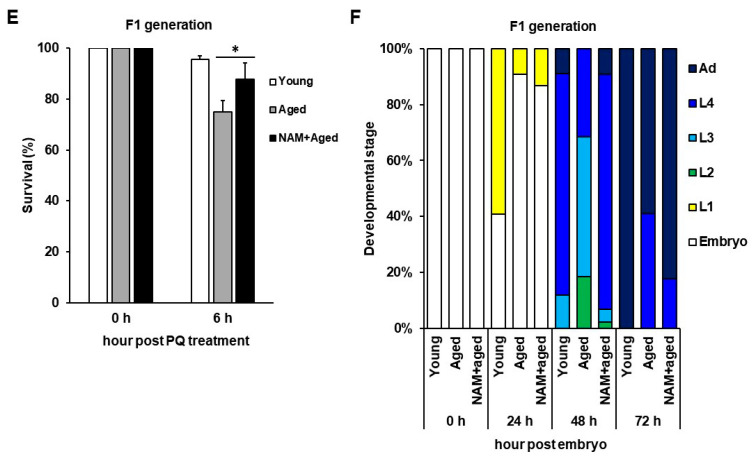
Motility, oxidative stress resistance, and developmental growth in offspring produced by aged oocytes are improved by maternal nicotinamide (NAM) supplementation in *Caenorhabditis elegans*: (**A**) The mitochondrial morphology was analyzed using SJ4103 expressing a mitochondrial-targeted GFP under the control of the muscle-specific *myo-3* promoter in the F1 transgenic animals produced by young, aged, and NAM+aged mothers (each condition, *n* = 40). The graph indicates the percentage of animals with muscle mitochondria classified into three categories—1) normal, 2) fused, and 3) fragmented; (**B**) The myofilament abnormality was visualized using the MYO-3::GFP transgene in the F1 generation of young, aged, and NAM+aged mothers. Representative fluorescence images of the F1 generation produced by young, aged, and NAM+aged mothers are shown (each condition, *n* = 40). The graph indicates the percentage of animals classified as normal or damaged muscle; (**C**) Comparison of head thrashing in the F1 generation produced by young, aged, and NAM+aged mothers (each condition, *n* = 30). Error bars represent standard deviation (SD). **, *p* < 0.01 (one-way ANOVA with Tukey’s post hoc test); (**D**) Comparison of body bending in the F1 generation of young, aged, and NAM+aged mothers (each condition, *n* = 30). Error bars represent SD. **, *p* < 0.01 (one-way ANOVA with Tukey’s post hoc test); (**E**) The percentage of survival rate in the F1 generation produced by young, aged, and NAM+aged mothers was analyzed under paraquat (100 mM)-induced oxidative stress condition (each condition, *n* = 30). Error bars represent SD. *, *p* < 0.05 (two-way ANOVA with Tukey’s post hoc test); (**F**) The developmental stage of each individual in the F1 generation produced by young, aged, and NAM+aged mothers was determined based on its size and stage-specific morphological characteristics (see Materials and Methods) during development.

We then determined whether maternal NAM supplementation in aged animals improved the mitochondrial function of the F1 generation. We analyzed the mitochondrial morphology and the integrity of muscle cells in the F1 generation produced by aged mothers with NAM supplementation by observing the expression of the transgene *myo-3::GFP(mit)* or *myo-3::GFP* in transgenic animals (Figure 5A,B). We found that maternal NAM supplementation improved both mitochondrial morphology and the integrity of muscle cells in the F1 generation compared to the F1 generation from non-treated aged mothers (Figure 5A,B). These findings suggest that NAM-induced improvement in the mitochondrial function in aged mothers is intergenerationally conserved in the F1 generation.

Abnormal mitochondria in muscle cells are associated with changes in locomotion behavior [46]. To investigate whether the improved muscle integrity in the F1 generation by maternal NAM supplementation affected the motility behavior, we measured the body bending rates and head thrashes in the F1 generation from young, non-treated aged, and NAM-treated aged mothers. Compared to those in the F1 generation from non-treated aged mothers, the body bending rates and head thrashes were significantly increased in the F1 generation from NAM-treated aged mothers (Figure 5C,D). These results suggest that maternal dysfunctional mitochondria affect the locomotion behavior of the subsequent generation, which was promoted by maternal NAM supplementation through improved maternal mitochondrial function.

Then, we examined whether the induced oxidative stress response and developmental growth rate in the offspring could be altered by maternal NAM supplementation in aged animals. We found that the F1 generation from NAM-treated aged mothers had a higher survival rate compared to the F1 generation from non-treated aged mothers upon exposure to 100 mM paraquat (Figure 5E). We further evaluated the developmental growth rate in the F1 generation from young, non-treated aged, and NAM-treated aged mothers. The F1 generation from non-treated aged mothers showed a significant decline in the developmental growth rate compared to the F1 generation from young mothers (Figure 5F). However, the developmental growth rate of the F1 generation from NAM-treated aged mothers was comparable to that of young mothers (Figure 5F), suggesting that maternal dysfunctional mitochondria in aged animals affect the developmental progress of the subsequent generation, which may be improved by maternal NAM supplementation.

Given the above findings that NAM improves oocyte quality by modulating the mitochondrial redox balance, we examined the effect of another antioxidant, N-acetyl-L-cysteine (NAC), on mitochondria in aged oocytes by Mitotracker and CellROX Green staining. We found that NAC improved not only the mitochondrial morphology and activity but also significantly reduced the age-induced mitochondrial ROS levels in aged oocytes (Supplementary Figure S6). These results imply that the decline in the quality of aged oocytes is primarily caused by dysfunctional mitochondria with imbalanced redox homeostasis, which can be restored by maternal antioxidant supplementation.

## 4. Discussion

Nicotinamide (NAM) is one of the major precursors of nicotinamide adenine dinucleotide (NAD^+^). NAD^+^ is synthesized through three pathways depending on the availability of the precursors, including tryptophan, nicotinic acid, nicotinamide riboside (NR), nicotinamide mononucleotide, and NAM [47]. As a central metabolic regulator, NAD^+^ is required for maintaining mitochondrial dynamics and is essential for tissue health [48,49,50,51]. The effects of different NAD+ precursors on the lifespan and healthspan of worms, flies, and mice have been investigated [36,52,53]. Interestingly, in *C. elegans*, NR supplementation extended the lifespan through the SIR-2.1 pathway [54]. The genetic overexpression of the NAD^+^ synthetic enzyme nicotinamidase (D-NAAM) also extended the lifespan of flies [52]. Consistently, in a mouse model, NR supplementation also extended the lifespan [53]. These findings suggest that the level of NAD^+^ decreases in an age-dependent manner; thus, supplementation with NAD^+^ or its precursor suppresses the aging process across species.

In the present study, we found that NAM supplementation improves reproductive capacity in aged animals by recovering mitochondrial function in aged oocytes. Consistent with previous studies [5,6], we also found that a decline in oocyte quality due to mitochondrial dysfunction and imbalanced redox homeostasis is a critical factor during reproductive aging. NAM supplementation showed an anti-aging effect on female reproductive aging, including a significant decrease in embryonic lethality, oocytes with abnormal chromosome morphology, and levels of RAD-51 foci during homologous recombination (HR) processes with age. How does NAM supplementation improve chromosome morphology and HR events? Several previous reports suggest that NAM-related supplementation improves genomic integrity [15]. The NAM-consuming enzyme, nicotinamide phosphoribosyltransferase, plays an important role in DNA repair to maintain genome integrity in muscle cells [35]. Additionally, NR supplementation improved genomic stability through the Sirtuin 1 pathway in both mouse and *C. elegans* models [6,54]. Based on previous findings and our results, we propose that NAM supplementation contributes to maintaining the chromosome integrity of aged oocytes by promoting repair processes during gametogenesis in aged mothers. The molecular mechanism involved in the contribution of NAM supplementation to DNA repair capacity in aged germ cells remains elusive.

Furthermore, we showed that NAM supplementation maintained the normal level of germ cell apoptosis in aged *C. elegans* mothers by decreasing the level of CED-9, a homolog of anti-apoptotic Bcl-2 but not CED-4, a homolog of Apaf-1. The BCL-2-like protein CED-9 of *C. elegans*, which is localized predominantly at the outer mitochondrial membrane, blocks apoptosis by inhibiting pro-apoptotic CED-4 [55,56,57]. In contrast to CED-9, CED-4 is not localized at the mitochondria; it is localized at the perinuclear region of germ cells in *C. elegans* [58]. CED-9 is known to control mitochondrial morphology and dynamics in *C. elegans*, which agrees with its location [59,60]. The high level of CED-9 in aged animals was lowered by NAM supplementation which may increase germ cell apoptosis, suggesting that the level of CED-9 is controlled by improving mitochondrial function in NAM-treated aged animals. Germ cell apoptosis controls oocyte quality by reducing the number of germ cells to ensure the proper allocation of resources during oogenesis [7,10]. This suggests that a normal level of germ cell apoptosis is required for proper reproduction. Furthermore, it was shown that mitochondria can exit from apoptotic germ cells and can directly enter into oocytes through a flow of transport in the gonad core [61]. Therefore, it appears that germ cell apoptosis is severely decreased to prevent the transport of defective mitochondria into oocytes in aged animals. This finding suggests that germ cell apoptosis regulates mitochondrial quality in oocytes. In our study, the quality of mitochondria in aged oocytes was improved by NAM supplementation, which may be due to the increased level of germ cell apoptosis and decreased level of CED-9. This speculation is supported by the results showing that embryonic lethality was significantly increased in apoptosis-defective *ced-3* mutants.

In mammals, the mitochondria are uniparentally transmitted through mothers [62]. Thus, maternal mitochondria are replicated during embryogenesis to colonize both somatic and germ cells in offspring [63]. Therefore, mitochondrial abnormalities present in oocytes are passed down, leading to developmental defects in offspring [64,65,66,67]. Reports suggest that mitochondrial dysfunction in oocytes increases the risk of metabolic diseases in offspring [65,68]. These facts highlight that mitochondrial activity in oocytes is critical for the health of offspring. Despite the importance of maternal mitochondria in offspring, it is largely unknown how to protect maternal mitochondria during reproductive aging. Here, we proposed that maternal NAM supplementation can contribute to the maintenance of mitochondrial function in aged oocytes as well as in offspring. Furthermore, we showed that maternal NAM supplementation significantly improved motility, oxidative stress response, and developmental growth.

Due to increasing maternal age in modern societies, the problem of infertility caused by reproductive aging is an emerging social issue. Therefore, it is important to explore the basis of female reproductive aging. Although no single treatment has been recommended for clinical application to improve in poor ovarian response patients [69], the present study suggests the possibility of the nutritional supplementation of a single compound such as NAM in improving the quality of aged oocytes. Based on our findings, we propose that NAM supplementation in the early stages of aged oocytes delays the aging process of oocyte and improves the aged oocyte quality. Overall, we provide in vivo evidence that maternal NAM supplementation improves mitochondrial function in aged oocytes and suppresses age-dependent excessive ROS generation, which delays reproductive aging and thus increases fertility. Furthermore, maternal NAM supplementation improves developmental growth and mitochondrial function in offspring produced by aged oocytes.

## 5. Conclusions

This study provides several evidence showing beneficial effects of nicotinamide (NAM) supplementation on the reproductive capacity in aged oocytes and the intergenerational effects of maternal NAM intake. These effects were attributed to the improved mitochondrial function and the chromosomal integrity in aged oocytes by NAM supplementation. These findings support that mother’s diet is critical for the oxidative stress resistance and developmental growth of offspring produced by aged oocytes.

## Figures and Tables

**Table 1:** Chromosome morphology at -1 oocyte (%).

	Aligned Condensed	Under- Condensed	Misaligned Condensed	*n*
Young	100	0	0	30
Aged	65.38	19.23	15.38	17
NAM+Aged	77.76	11.13	11.11	21

## Data Availability

Not applicable.

## Supplementary Materials

The following are available online at https://www.mdpi.com/article/10.3390/antiox10040519/s1.
