# Supplementary figures and images for: Nicotinamide Supplementation Improves Oocyte Quality and Offspring Development by Modulating Mitochondrial Function in an Aged Caenorhabditis elegans Model
Source: Antioxidants (Basel). 2021 Mar 26;10(4):519. doi: 10.3390/antiox10040519 (PMC8066965; doi:10.3390/antiox10040519)

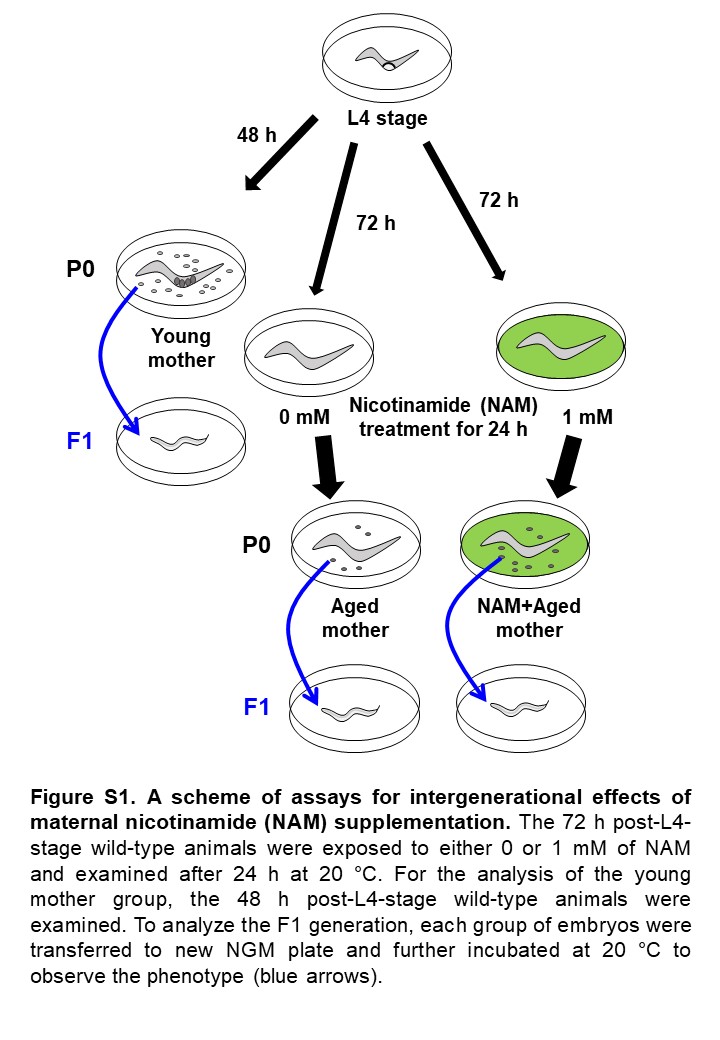

Supplement: Supplementary file 1 [file antioxidants-10-00519-s001.zip › Supplementary Figure S1.jpg]

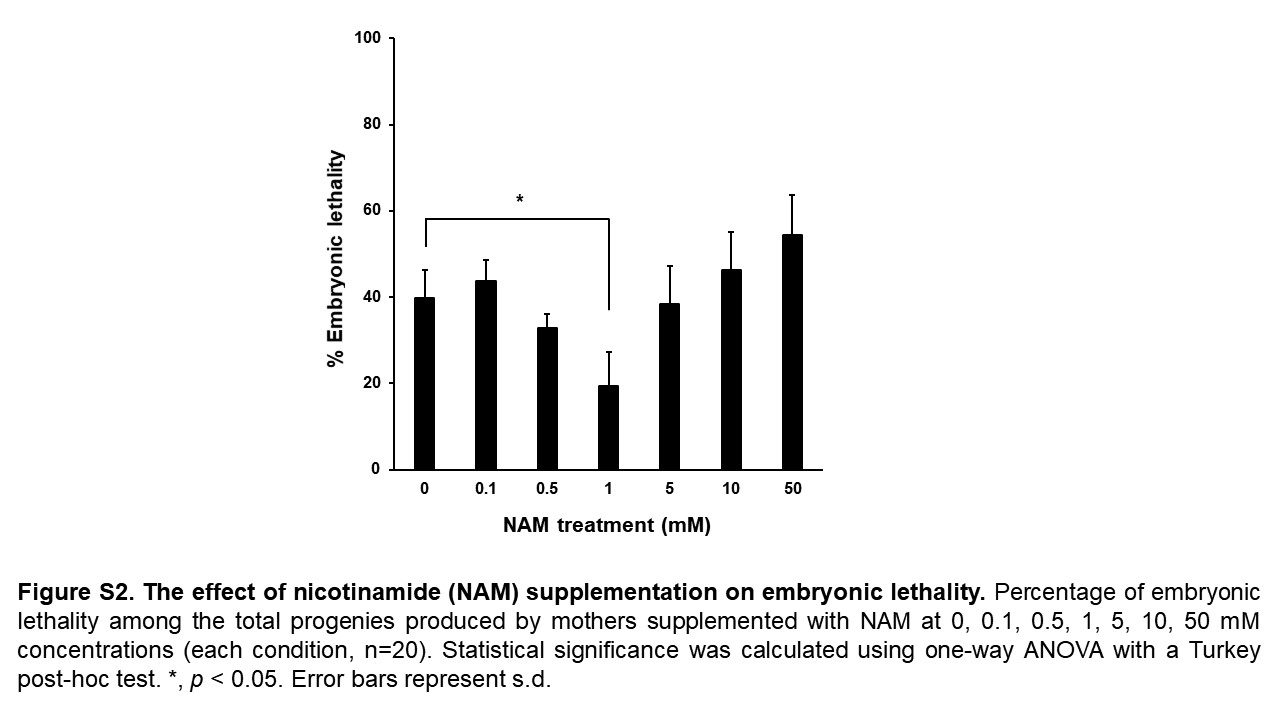

Supplement: Supplementary file 1 [file antioxidants-10-00519-s001.zip › Supplementary Figure S2.JPG]

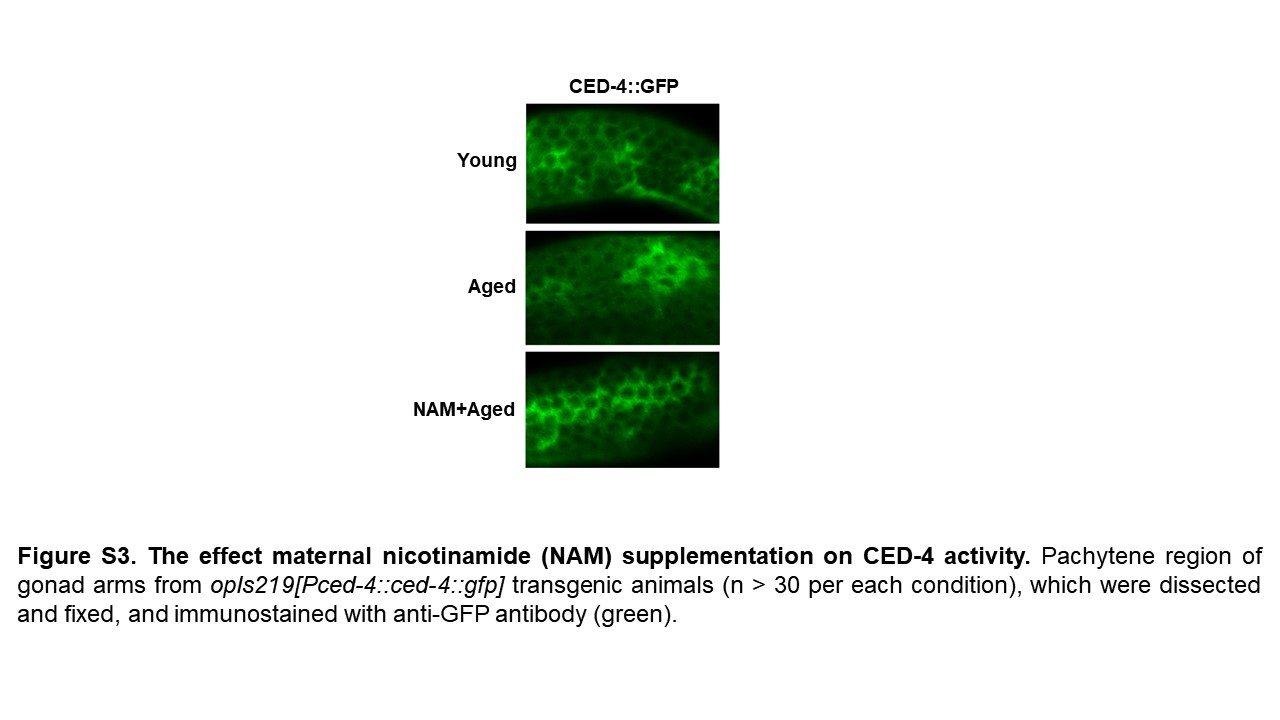

Supplement: Supplementary file 1 [file antioxidants-10-00519-s001.zip › Supplementary Figure S3.JPG]

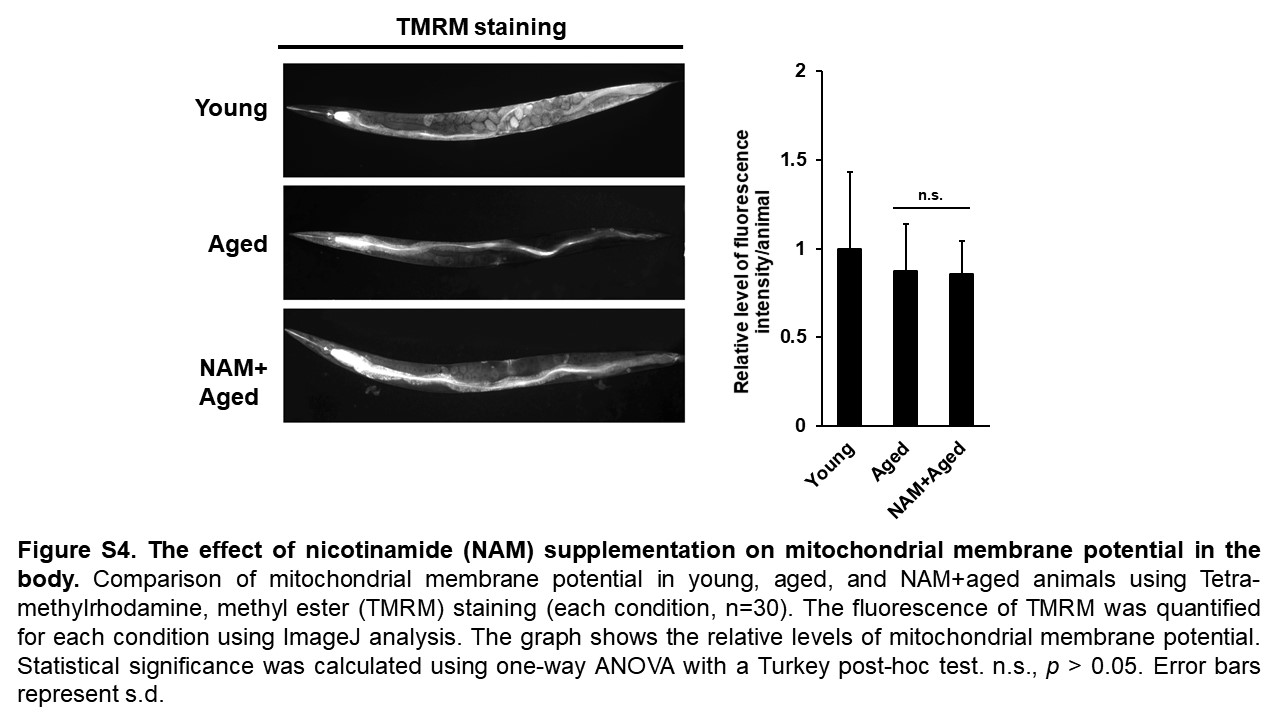

Supplement: Supplementary file 1 [file antioxidants-10-00519-s001.zip › Supplementary Figure S4.JPG]

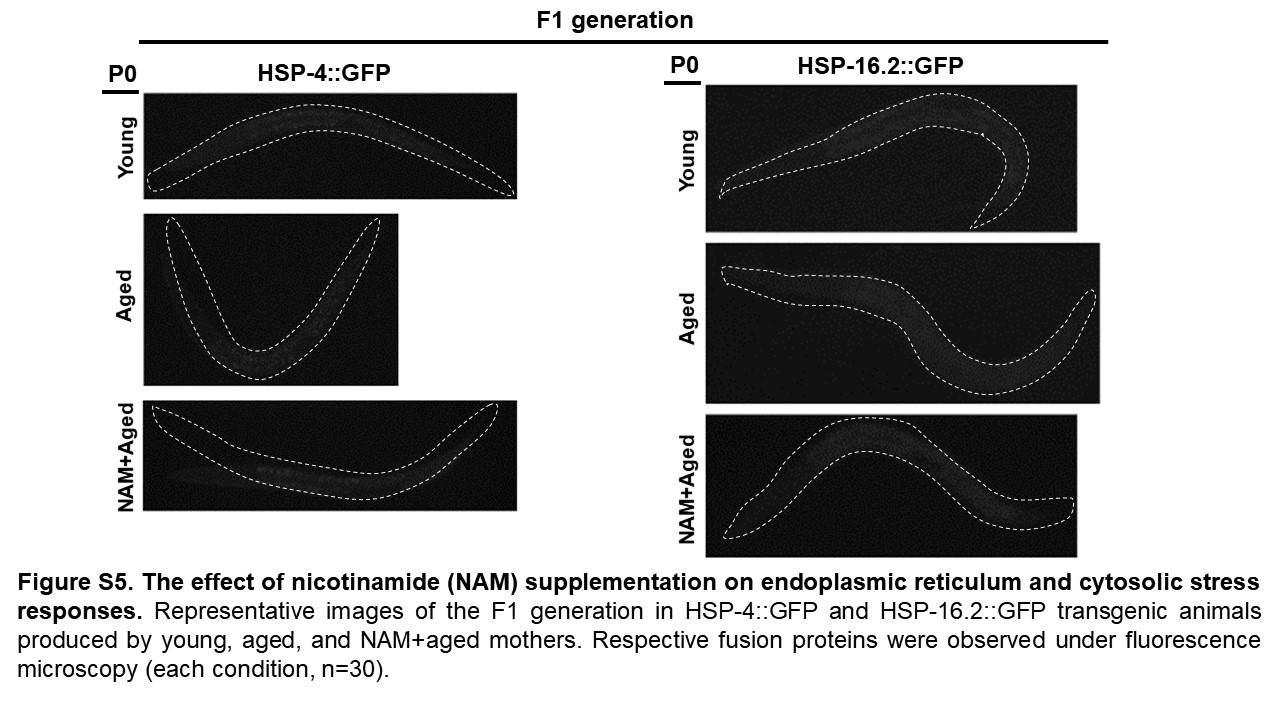

Supplement: Supplementary file 1 [file antioxidants-10-00519-s001.zip › Supplementary Figure S5.JPG]

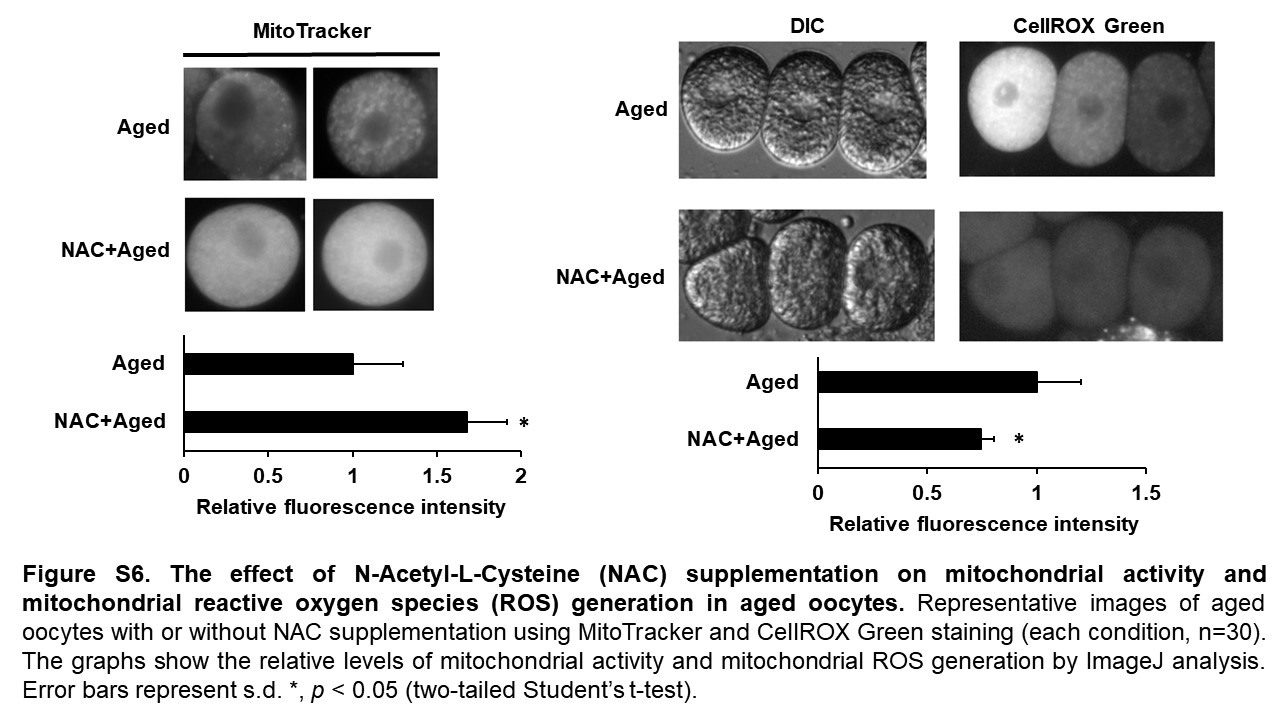

Supplement: Supplementary file 1 [file antioxidants-10-00519-s001.zip › Supplementary Figure S6.JPG]
